# Fentanyl as an induction agent for tracheal intubation in critically ill patients: a systematic review and meta-analysis

**DOI:** 10.1186/s40560-026-00866-7

**Published:** 2026-02-07

**Authors:** Yuki Kotani, Takatoshi Koroki, Takeshi Nomura, Yoshiro Hayashi

**Affiliations:** 1 Department of Intensive Care Medicine, Tokyo Women’s Medical University, 8-1 Kawada-cho, Shinjuku-ku, Tokyo, 162-8666 Japan; 2 Department of Intensive Care Medicine, Kameda Medical Center, 929 Higashi-Cho, Kamogawa, Chiba 296-8602 Japan

**Keywords:** Systematic review, Fentanyl, Intubation, Critical illness, Hypotension

## Abstract

**Background:**

Tracheal intubation in critically ill adults is frequently complicated by severe physiological adverse events, particularly cardiovascular instability. Although fentanyl is commonly used for induction, observational data suggest that its use may increase the risk of post-intubation hypotension. However, the overall randomized evidence remains unclear. In this systematic review and meta-analysis of randomized controlled trials (RCTs), we hypothesized that induction regimens including fentanyl or its analogs would increase the risk of peri-intubation cardiovascular instability in critically ill patients.

**Methods:**

We comprehensively searched PubMed, Embase, the Cochrane Library, ClinicalTrials.gov, and the WHO ICTRP from inception through October 31, 2025. Eligible studies were RCTs comparing an induction regimen including fentanyl or its analogs with one without them in critically ill adults undergoing tracheal intubation. The primary outcome was peri-intubation cardiovascular instability. Secondary outcomes included peri-intubation hypoxemia, successful intubation on the first attempt, duration of mechanical ventilation, ICU length of stay, and mortality. Random-effects meta-analyses were performed for all outcomes. Trial sequential analysis (TSA) was conducted for the primary outcome. Certainty of evidence was assessed using the GRADE approach.

**Results:**

We included five RCTs and 515 participants. Two studies were judged to be low risk of bias, two raised some concerns, and one was at high risk of bias. Comparators included various induction agents and placebo. Definitions of peri-intubation cardiovascular instability also varied. The evidence was very uncertain regarding the effect of fentanyl on the risk of peri-intubation cardiovascular instability (risk ratio, 1.41; 95% confidence interval, 0.83–2.40; risk difference, 9.2% more; 95% confidence interval, 3.8% fewer to 31.3% more; very low certainty). In TSA, the required information size (*n* = 5586) was not reached, indicating the lack of statistical power. The certainty of evidence for pooled secondary outcomes was generally low or very low.

**Conclusions:**

The effect of fentanyl on peri-intubation cardiovascular instability remains highly uncertain, with pooled estimates compatible with substantial harm, substantial benefit, or no effect. Current randomized evidence is insufficient to guide routine clinical practice, given their very low or low certainty and susceptibility to random error.

**Trial registration:**

PROSPERO (registration number: CRD420251241214).

**Supplementary Information:**

The online version contains supplementary material available at 10.1186/s40560-026-00866-7.

## Introduction

Tracheal intubation in critically ill patients frequently results in severe adverse events, including hemodynamic instability, which are associated with an increased risk of mortality [1, 2]. Induction with analgesics and hypnotics is essential to facilitate intubation yet may precipitate cardiovascular collapse by blunting sympathetic tone [3]. To date, randomized evidence has been insufficient to guide optimal the selection of optimal induction regimens, leaving clinicians to rely on experience or local protocols [4].

Fentanyl is commonly incorporated into induction regimens in critically ill patients [1, 5, 6]. Although its hemodynamic effects during peri-intubation remain poorly understood, observational data have suggested an association between its use and post-intubation hypotension in emergency settings [7, 8], raising concerns given the established association between cardiovascular instability and mortality [2]. While several randomized controlled trials (RCTs) have evaluated fentanyl or its analogs in emergency intubation, these studies were small, heterogeneous in dosing and co-administered agents, and yielded inconsistent findings [9, 10]. Recent consensus statements and trials have emphasized hemodynamic-sparing peri-intubation strategies [4, 11, 12], therefore, clarifying the randomized evidence on fentanyl is increasingly important.

Despite its clinical importance, no systematic review has synthesized the available RCT evidence. The only previous review addressing adjunctive fentanyl added to a ketamine-based induction regimen identified a single observational study, leaving the overall certainty and applicability of the randomized evidence unclear [13]. This knowledge gap underscores the need for a comprehensive evaluation of RCTs assessing fentanyl during induction in critically ill adults.

Accordingly, we performed a systematic review and meta-analysis of RCTs to test the hypothesis that induction regimens including fentanyl or its analogs, compared with induction without these agents, would increase the risk of peri-intubation cardiovascular instability in critically ill adult patients undergoing tracheal intubation.

## Methods

We followed the PRISMA 2020 statement [14] and registered the review protocol in PROSPERO (registration number: CRD420251241214) (the PRISMA 2020 checklist is included in the Additional file 1). We selected the eligible studies based on the following PICOS framework: in critically ill adult patients undergoing tracheal intubation, does an induction regimen including fentanyl or its analogs, compared with a regimen without them, increase the risk of peri-intubation cardiovascular instability in RCTs?

### Search strategy and study selection

Two investigators (YK and TK) independently searched PubMed, Embase, the Cochrane Library, ClinicalTrials.gov, and the WHO ICTRP from inception through October 31, 2025, without language restrictions. We included RCTs comparing an induction regimen including fentanyl or its analogs with one without these agents in critically ill adults undergoing tracheal intubation. The analogs of fentanyl included sufentanil, alfentanil, remifentanil, and carfentanil. Critically ill adults undergoing tracheal intubation were defined as patients aged ≥ 18 years who underwent tracheal intubation for acute illness in an emergency setting. Exclusion criteria were pediatric populations (< 18 years), adult patients receiving tracheal intubation for an elective surgery or procedure, fentanyl or its analogs used both in the intervention and control groups, non-randomized studies, quasi-randomized studies, reviews, commentaries, editorials, and congress abstracts. We also excluded studies identified in clinical trial registries (i.e., ClinicalTrials.gov or WHO ICTRP) but not accompanied by a full-text publication, because the lack of full-text publication precludes adequate assessment of the risk of bias. The complete search strategies are available in the Additional file 1.

After de-duplication, two investigators (YK and TK) independently screened titles and abstracts and assessed full-text reports. Disagreements were resolved by discussion or adjudication by a third investigator (YH).

### Data collection

Two investigators (YK and TK) independently extracted data using a standardized form, with discrepancies resolved by consensus or adjudication by a third author (YH). Extracted variables included first author name, publication year, number of patients, number of centers, country, the setting of tracheal intubation, induction regimen in the intervention and control groups (e.g., type and dose of drugs, and timing of drug administration), co-interventions (other drugs included in the induction regimen, peri-intubation hemodynamic management, and preoxygenation techniques), and study outcomes. We also collected the definition and observation timing of each outcome. Trial authors were contacted when data were missing.

### Outcomes

The primary outcome of this review was peri-intubation cardiovascular instability. Its definition was based on each trial, including hypotension with an absolute threshold, a reduction in blood pressure from baseline, or initiation or dose increase of vasopressors during induction or early post-intubation. Secondary outcomes were peri-intubation hypoxemia (defined by each trial), successful intubation on the first attempt, duration of mechanical ventilation, ICU length of stay, and mortality at the longest follow-up.

### Risk of bias and certainty of evidence

We assessed the risk of bias using the Cochrane Risk of Bias 2 (RoB 2) tool [15]. We graded the certainty of the evidence with the GRADE approach and generated a summary of findings table using GRADEpro software [16]. We contextualized results using GRADE narrative statements (high certainty = no qualifiers, moderate certainty = “probably”, low certainty = “may”, and very low certainty = an uncertain effect) [17]. For imprecision assessments, we used the null effect as the threshold for all dichotomous outcomes. For duration of mechanical ventilation and ICU length of stay, we used a threshold of 1 day.

We planned to assess small-study effects using visual inspection of funnel plots and Egger’s test when ≥ 10 studies were available [18].

### Statistical analysis

We pooled dichotomous outcomes as risk ratio (RR) using Mantel–Haenszel random-effects models and continuous outcomes as mean differences (MD) using inverse-variance random-effects models, each with 95% confidence intervals (CIs). For dichotomous outcomes, we also presented absolute risk difference (RD). Between-study heterogeneity was quantified with Tau^2^ and *I*^2^. Two-sided *P* values < 0.05 was considered statistically significant.

We conducted two subgroup analyses based on the type of intervention (i.e., fentanyl vs. any of its analogs) and comparator (placebo vs. non-placebo drug). We also performed a sensitivity analysis restricted to studies judged to be at overall low risk of bias. These subgroup and sensitivity analyses were applied to all outcomes. In addition, to evaluate how heterogeneity in outcome definition affected the treatment effects, we performed a subgroup analysis of the primary outcome based on the definition (blood pressure thresholds vs. therapeutic interventions). These analyses were performed using Review Manager 5.4.

To assess the robustness of the pooled primary outcome data, we performed a trial sequential analysis (TSA). We calculated the diversity-adjusted information size under assumptions of a two-sided alpha = 0.05, power = 0.8, and a 25% risk ratio increase. We used the average event rate in the control group as the baseline risk. Analyses were performed using TSA Viewer (version 0.9.5.10 Beta. Copenhagen Trial Unit, Centre for Clinical Intervention Research, Rigshospitalet, Copenhagen, Denmark).

## Results

**Fig. 1:**
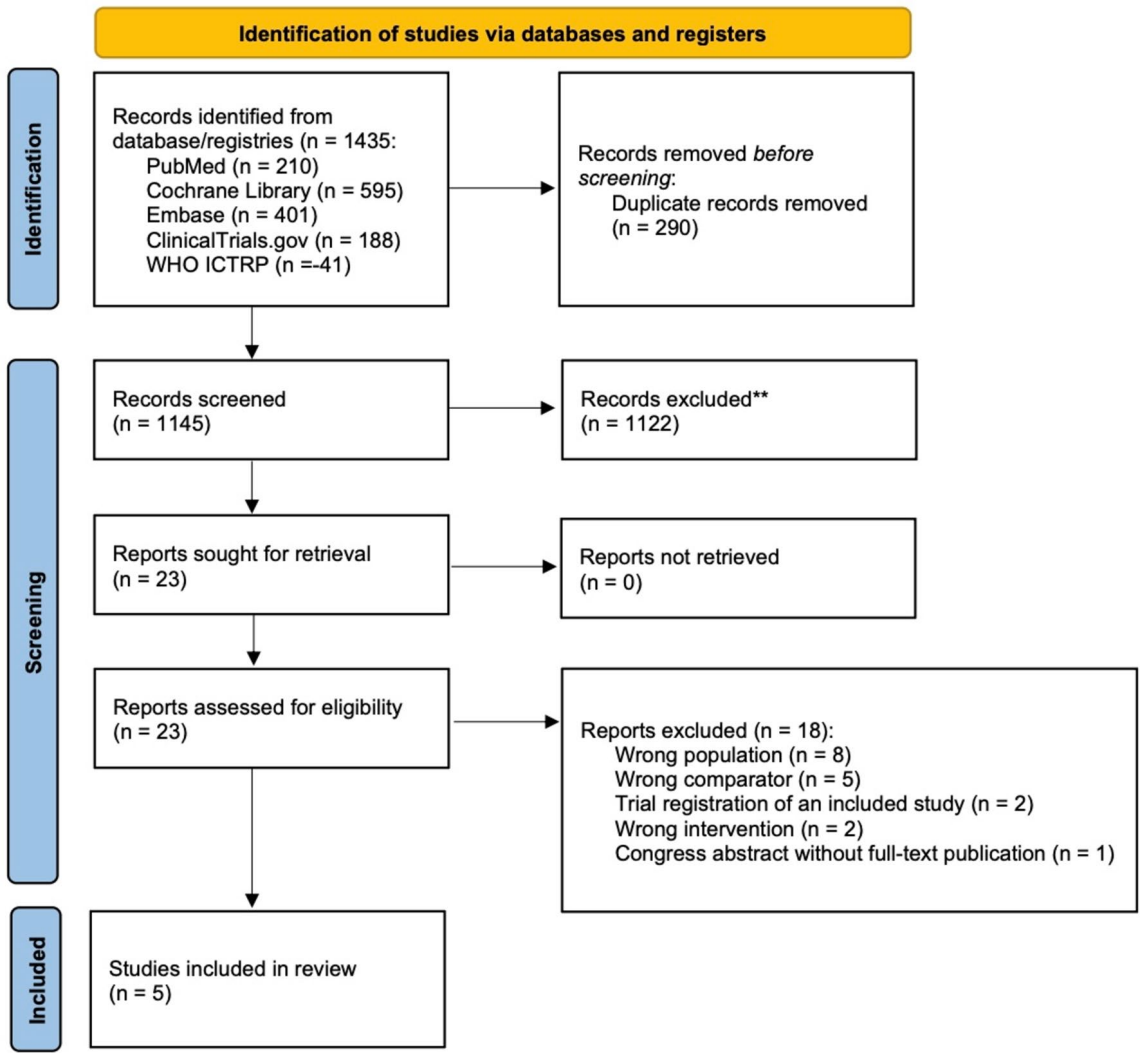
PRISMA 2020 flow diagram

We identified 1435 records through database and registry searches, and screened 1145 titles/abstracts after removing 290 duplicates. After excluding 1122 studies, we assessed 23 full-text reports for eligibility, and 18 were excluded. Finally, five RCTs (*n* = 515) were included (Fig. 1) [9, 10, 19–21]. Full-text exclusions and reasons for exclusions are detailed in Additional file 1: Table S1. Risk of bias was judged to be low in two studies [9, 21], to raise some concerns in two [19, 20], and to be high in one [10] (Additional file 1: Table S2).

### Study characteristics

Among the included studies, three studies were conducted in the emergency department [9, 19, 21], one in trauma center [20], and one in the ICU [10]. Four studies were single-center (*n* = 30–86) [10, 19–21] and one was multicenter (n = 291) [9]. A double-blind design was used in four studies [9, 10, 19, 21] and an open-label design was used in one [20].

The intervention was fentanyl in four studies (weight-based 0.5–5 µg/kg or fixed 100 µg) [9, 19–21] and sufentanil 1.5 µg/kg in one [10]. Comparators were placebo in one study [9] and other induction agents in four [10, 19–21]. All studies used neuromuscular blockade agents (succinylcholine or rocuronium) [9, 10, 19–21].

Table 1 also shows the definition of peri-intubation cardiovascular instability in each study. The definition used a blood pressure threshold in three studies [9, 10, 21] and a therapeutic intervention in two studies [19, 20]. The observation window was within 10 min in two studies [9, 21], 1 h in two studies [10, 19], and 24 h in one study [20].

**Table 1:** Study characteristics

Study	Setting	No. of centers	No. of patients	Design	Intervention	Control	Other induction agents	Definition of peri-intubation cardiovascular instability
Sivilotti 1998^a^	ED	1	86	Double-blind	Fentanyl 5 µg/kg	Midazolam 0.1 mg/kg or Thiopental 5 mg/kg	All groups: succinylcholine 1.5 mg/kg	Need for specific treatment within 1 h of intubation
Hildreth 2009	Trauma center	1	30	Open-label	Fentanyl 100 µg	Etomidate 0.3 mg/kg	Intervention group: midazolam 5 mgBoth groups: succinylcholine 1 mg/kg	Receipt of vasopressors within 24 h after induction
Ali 2021	ED	1	42	Double-blind	Fentanyl 2.5 µg/kg	Ketamine 1 mg/kg	Both groups: midazolam 0.05 mg/kg and succinylcholine 1 mg/kg	MAP ≤ 80% the baseline value within 10 min of induction
Ferguson 2022	ED	5	291	Double-blind	Fentanyl 0.5–2 µg/kg^b^	Placebo	Both groups: ketamine 0.5–2 mg/kg and rocuronium 1.5 mg/kg	SBP < 100 mmHg within 10 min of induction
Zhang 2025	ICU	1	80	Double-blind	Sufentanil 1.5 µg/kg	Esketamine 0.5–1 mg/kg	Intervention group: midazolam 40 µg/kgBoth groups: rocuronium 0.6 mg/kg	SBP < 90 mmHg during induction and within 1 h after intubation

^a^3-arm study.

^b^The dose of fentanyl was 1/1000 of that of ketamine, which was determined by the treating physician.

ED, emergency department; ICU, intensive care unit; MAP, mean arterial pressure; NIV, noninvasive ventilation; SBP, systolic blood pressure

Peri-intubation hemodynamic management is summarized in Additional file 1: Table S3. Norepinephrine infusion was used in response to cardiovascular instability in two studies [10, 21]. Another study used fluid boluses and inotropes without specified initiation criteria. No specific protocol was reported in two studies [19, 20].

### Primary outcome

**Fig. 2:**
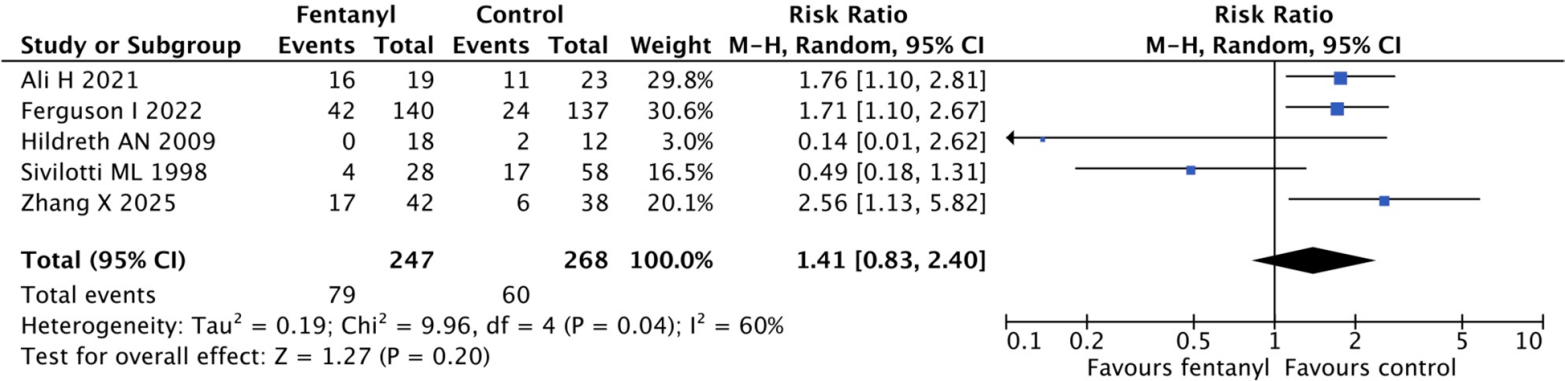
Forest plot of peri-intubation cardiovascular instability

The pooled effect on peri-intubation cardiovascular instability was very uncertain (RR, 1.41; 95% CI, 0.83 to 2.40; RD, 9.2% more; 95% CI, 3.8% fewer to 31.3% more; very low certainty) (Fig. 2, Tables 2, 3). Subgroup analyses by intervention (fentanyl vs. analogue) and comparator (placebo vs. non-placebo) did not detect a significant interaction (P for interaction = 0.14 and 0.33, respectively; Table 2, Additional file 1: Figures S1 and S2).

**Table 2:** Sensitivity analysis of peri-intubation cardiovascular instability

Subgroup	No. of studies	Fentanyl	Control	Risk ratio (95% CI)	*P* value	*I*^2^	*P* for interaction
Overall	5	79/247 (32%)	60/268 (22%)	1.41 (0.83 to 2.40)	0.20	60%	
Type of intervention							0.14
Fentanyl	4	62/205 (30%)	54/230 (23%)	1.18 (0.62 to 2.24)	0.61	66%	
Any analogue	1	17/42 (40%)	6/38 (16%)	2.56 (1.13 to 5.82)	0.02	NA	
Type of comparator							0.33
Placebo	2	58/159 (36%)	35/160 (22%)	1.74 (1.26 to 2.39)	< 0.001	0%	
Non-placebo drug	3	21/88 (24%)	25/108 (23%)	0.79 (0.17 to 3.65)	0.77	77%	
Outcome definition							0.004
Blood pressure thresholds	3	75/201 (37%)	41/198 (21%)	1.83 (1.35 to 2.47)	< 0.001	0%	
Therapeutic interventions	2	4/46 (8.7%)	19/70 (27%)	0.43 (0.17 to 1.10)	0.08	0%	
Low risk-of-bias studies	2	58/159 (36%)	35/160 (22%)	1.74 (1.26 to 2.39)	< 0.001	0%	

CI, confidence interval; NA, not applicable.

**Table 3:** Summary of finding’s table

Certainty assessment							№ of patients		Effect		Certainty	Importance
№ of studies	Study design	Risk of bias	Inconsistency	Indirectness	Imprecision	Other considerations	fentanyl	control	Relative (95% CI)	Absolute (95% CI)		
**Peri-intubation cardiovascular instability**												
5	Randomised trials	Not serious	Serious^a^	Serious^b^	Serious^c^	None	79/247 (32.0%)	60/268 (22.4%)	**RR 1.41**(0.83 to 2.40)	**92 more per 1,000** (from 38 fewer to 313 more)	⨁◯◯◯ Very low^a,b,c^	CRITICAL
**Peri-intubation hypoxemia**												
2	Randomised trials	Not serious	Serious^a^	Not serious	Serious^c^	None	32/171 (18.7%)	36/206 (17.5%)	**RR 0.93**(0.31 to 2.83)	**12 fewer per 1,000**(from 121 fewer to 320 more)	⨁⨁◯◯Low^a,c^	CRITICAL
**Successful intubation on the first attempt**												
1	Randomised trials	Not serious	Not serious	Not serious	Serious^c^	None	132/140 (94.3%)	136/148 (91.9%)	**RR 1.03**(0.96 to 1.09)	**28 more per 1,000**(from 37 fewer to 83 more)	⨁⨁⨁◯Moderate^c^	CRITICAL
**Duration of mechanical ventilation**												
3	Randomised trials	Not serious	serious^a^	Not serious	Serious^c^	None	197	204	–	MD **0.19 day more**(4.29 fewer to 4.66 more)	⨁⨁◯◯Low^a,c^	IMPORTANT
**ICU length of stay**												
2	Randomised trials	Serious^d^	Serious^a^	Not serious	Serious^c^	None	54	56	–	MD **0.93 day more**(11.12 fewer to 12.98 more)	⨁◯◯◯Very low^a,c,d^	IMPORTANT
**Mortality at the longest follow-up**												
3	Randomized trials	Serious^e^	Not serious	Not serious	Serious^c^	None	38/204 (18.6%)	46/198 (23.2%)	**RR 0.84**(0.57 to 1.23)	**37 fewer per 1,000**(from 100 fewer to 53 more)	⨁⨁◯◯Low^c,e^	CRITICAL

CI, confidence interval; MD, mean difference; RR, risk ratio.

^a^Results vary in magnitude and direction across trials.

^b^The definition of this outcome varied across studies.

^c^The confidence interval was wide confidence interval and the sufficient information size was not met.

^d^Methodological limitations in both small trials.

^e^Timing of assessing mortality varied across studies.

When stratified by outcome definition (i.e., blood pressure thresholds vs. therapeutic interventions), an interaction was observed (P for interaction = 0.004). In blood pressure threshold-defined studies, fentanyl may increase peri-intubation cardiovascular instability (RR, 1.83; 95% CI, 1.35 to 2.47; RD, 17.2% more; 95% CI, 7.2% more to 30.4% more; low certainty) (Table 2, Additional file 1: Figure S3). In therapeutic intervention-based studies, the pooled effect was very uncertain (RR, 0.43; 95% CI, 0.17 to 1.10; RD, 15.5% fewer, 95% CI, 22.5% fewer to 2.7% more) (Table 2, Additional file 1: Figure S3).

A sensitivity analysis restricted to overall low risk-of-bias studies suggested that fentanyl may increase peri-intubation cardiovascular instability (RR, 1.74; 95% CI, 1.26 to 2.39; RD, 16.2% more; 95% CI, 5.7% more to 30.4% more; low certainty; Additional file 1: Figure S4). All subgroup and sensitivity analyses were not adjusted for multiplicity.

In TSA, the required information size was not reached (*n* = 5586), indicating that current randomized evidence remains underpowered to draw firm conclusions (Additional file 1: Figure S5).

### Secondary outcomes

Fentanyl may have little to no effect on peri-intubation hypoxemia (RR, 0.93; 95% CI, 0.31 to 2.83; RD, 1.2% fewer; 95% CI, 12% fewer to 32% more; low certainty; Additional file 1: Figure S6) and duration of mechanical ventilation (3 studies; MD, 0.19 days more; 95% CI, 4.29 fewer to 4.66 more; low certainty; Additional file 1: Figure S7). Fentanyl probably has little to no effect on successful intubation on the first attempt (RR, 1.03; 95% CI, 0.96 to 1.09; RD, 2.8% more; 95% CI, 3.7% fewer to 8.3% more; moderate certainty; Additional file 1: Figure S8). Evidence for ICU length of stay was very uncertain (MD, 0.93 days; 95% CI, − 11.12 to 12.98; very low certainty; Additional file 1: Figure S9). Mortality at the longest follow-up may be decreased (RR, 0.84; 95% CI, 0.57 to 1.23; RD, 3.7% fewer; 95% CI, 10.0% fewer to 5.3% more; low certainty of evidence; Additional file 1: Figure S10). Timing of assessing mortality is summarized in Additional file 1: Table S4. The pooled analysis results for all outcomes are summarized in the summary of findings table (Table 3).

While most sensitivity analyses yielded similar findings to the main analysis, statistically significant interactions were found between the type of intervention (fentanyl vs. any analogue) and duration of mechanical ventilation and ICU length of stay, and between the type of comparator (placebo vs. non-placebo drug) and peri-intubation hypoxemia, duration of mechanical ventilation, and ICU length of stay (Additional file 1: Table S5). All subgroup and sensitivity analyses were not adjusted for multiplicity.

## Discussion

### Key findings

This systematic review identified five RCTs investigating fentanyl as an induction agent for tracheal intubation in critically ill adults. We observed heterogeneity in fentanyl dosing, comparator agents, peri-intubation hemodynamic care, and definitions of cardiovascular instability. These differences indicate that the studies operationalized the underlying physiological construct in distinct ways, which contributed to variability in effect estimates. Consequently, although a pooled estimate could be generated, the certainty of evidence was rated very low, and the true effect of fentanyl on peri-intubation hemodynamics remains uncertain.

### Comparison with previous literature

To our knowledge, no previous systematic review has synthesized randomized evidence on fentanyl for induction in critically ill adults. The only previous review examined adjunctive fentanyl in ketamine-based induction but identified only a single observational study, offering no insight into randomized evidence [13]. Our review, therefore, provides the first comprehensive assessment of the totality of randomized data on this topic. A central limitation we identified is construct heterogeneity in the primary outcome. The included studies operationalized cardiovascular instability using blood pressure thresholds or clinician-triggered vasopressor initiation. This heterogeneity limits interpretability of pooled estimates and aligns with broader variability in hemodynamic endpoints reported in intubation studies of critically ill adults. Development of a consensus, patient-centered definition and protocolized hemodynamic management would improve comparability across future trials.

The overall randomized evidence provides very low certainty regarding the hemodynamic effect of fentanyl. While the point estimate favoring control was in line with previous observational studies in the emergency settings [7, 8], the context dependence of the hemodynamic effects of fentanyl, driven by patient physiology, dose, and co-administered hypnotics (e.g., ketamine, midazolam, and etomidate), likely contributed to divergent study results. Comparator choice may also influence effect direction. Given that most studies used active comparators and only one study employed placebo, the observed results may reflect relative rather than absolute hemodynamic effects of fentanyl. Although subgroup interaction by comparator was not significant, the pattern was pharmacologically plausible and hypothesis-generating, given that any analgesics and sedatives can induce cardiovascular instability through vasodilation and/or reduced cardiac contractility [22]. Different clinical settings (i.e., emergency department, trauma center, and ICU) may also have contributed to heterogeneity in the observed effects because of the differences in baseline illness severity, resuscitation status, and staffing models. A sensitivity analysis restricted to low risk-of-bias studies suggested potential harm; however, between-study variability in dose, small sample sizes, and the underpowered TSA leave equipoise regarding the net hemodynamic effect of fentanyl.

For other secondary outcomes, the certainty of evidence was generally low or very low, primarily due to substantial imprecision and small sample sizes, which prevented confident estimation of treatment effects. Although several subgroup analyses yielded statistically significant findings, these results, including subgroup interactions, should not be overinterpreted and should be viewed as hypothesis-generating, because the analyses were underpowered and not adjusted for multiplicity.

### Implications for clinical practice and future research

Current evidence does not provide a reliable estimate of how fentanyl influences peri-intubation hemodynamics in critically ill adults. Given this uncertainty, fentanyl should neither be routinely avoided nor assumed to be hemodynamically safe. Rather, its use should be individualized based on a patient’s physiological reserve, pre-existing shock, anticipated sympathetic response, and the hemodynamic profile of co-administered hypnotic agents. Particular caution is warranted in patients with ongoing cardiovascular instability or limited cardiovascular reserve.

The apparent mortality reduction should not be interpreted as a true benefit and should not inform clinical decision-making, because it is based on few events, wide CIs, and no clear biological rationale, and is, therefore, most consistent with random variation.

This review highlights several methodological gaps that future trials should address. First, there was substantial heterogeneity in both intervention dosing (weight-based 0.5–5 µg/kg or fixed 100 µg) and comparator agents (placebo, midazolam, thiopental, etomidate), underscoring the need to define standardized fentanyl dosing strategies and clinically relevant comparators. Second, hemodynamic management protocols differed or were incompletely reported, potentially influencing outcome incidence. Third, outcome definitions varied across trials, using either absolute blood pressure thresholds or clinician-triggered vasopressor initiation, which represent distinct physiological contexts and limit comparability. Given the variations in outcome reporting, we suggest developing a core outcome set for intubation research in critically ill patients, which should include both physiological endpoints and patient-centered outcomes, to improve trial comparability and facilitate evidence synthesis.

Future research should incorporate a protocolized peri-intubation hemodynamic strategy, adopt a consensus definition of cardiovascular instability, and ensure adequate sample size to overcome current imprecision. An adequately powered, multicenter RCT using harmonized dosing, standardized co-interventions, and patient-centered hemodynamic outcomes would meaningfully advance the evidence base and help define the appropriate role of fentanyl during induction in critically ill adults.

### Strengths and limitations

This study represents the first systematic review and meta-analysis of randomized trials evaluating fentanyl as an induction agent for tracheal intubation in critically ill adults. This review was conducted according to a pre-registered protocol and adhered to contemporary methodological standards. Beyond synthesizing the totality of randomized evidence, this study also clarifies several methodological shortcomings in previous trials, particularly heterogeneity in outcome definitions and co-interventions, which helps inform the design of future RCTs.

Several limitations merit consideration. First, only five, relatively small studies were eligible, limiting the precision of pooled estimates and precluding definitive conclusions.

Second, the definition of peri-intubation cardiovascular instability varied across studies, particularly between blood pressure threshold-based and therapeutic intervention-based outcomes. Pooling outcomes defined by fundamentally different criteria may compromise the interpretability of the synthesized estimates. To assess the potential impact of this heterogeneity, we conducted a subgroup analysis according to outcome definition and observed a significant interaction. This limitation underscores the need for cautious interpretation of pooled results and highlights the importance of developing a standardized consensus definition for this clinically relevant outcome.

Third, both the intervention dose and comparator agents varied considerably across studies. Although we performed sensitivity analyses based on comparator type, the limited number of studies prevented more robust approaches, such as network meta-analysis. Fourth, the certainty of evidence was generally low to very low, largely due to imprecision, and unclear or high risk of bias in several studies. Future trials would benefit from standardized hemodynamic protocols, harmonized outcome definitions, and rigorous blinding to improve internal validity and between-study comparability.

## Conclusions

In this systematic review of five randomized trials, the effect of fentanyl on peri-intubation cardiovascular instability in critically ill adults remains highly uncertain. This uncertainty was driven primarily by heterogeneity in the operational definition of peri-intubation cardiovascular instability, which limits the interpretability of pooled estimates, in addition to small sample sizes and variations in dosing strategies, comparator agents, and hemodynamic management protocols. TSA indicated the inconclusiveness of our findings due to the lack of statistical power. Secondary outcomes, including mortality, should not be overinterpreted given their very low to low certainty and susceptibility to random error. Until more definitive evidence becomes available, the hemodynamic safety of fentanyl during induction should be considered unresolved. Well-powered, rigorously designed multicenter RCTs using standardized co-interventions and consensus outcome definitions are needed to clarify the role of fentanyl in this setting.

## Supplementary Information

**Additional file 1.**

## Data Availability

We collected the summary data from published randomized trials. All data generated or analyzed for this study are included in this published article and its supplementary files. Further information is available from the corresponding authors upon reasonable request.

## Abbreviations

ICU: Intensive care unit
CI: Confidence interval
GRADE: Grading of Recommendations Assessment, Development and Evaluation
PRISMA: Preferred Reporting Items for Systematic Reviews and Meta-Analyses
RR: Risk ratio
MD: Mean difference
