# Supplementary material for: Fentanyl as an induction agent for tracheal intubation in critically ill patients: a systematic review and meta-analysis
Source: J Intensive Care. 2026 Feb 7;14:25. doi: 10.1186/s40560-026-00866-7 (PMC12977560; doi:10.1186/s40560-026-00866-7)
Supplement: Supplementary file 1 — Additional file 1. [file 40560_2026_866_MOESM1_ESM.docx]

**Supplementary materials**

**Fentanyl as an induction agent for tracheal intubation in critically ill patients: a systematic review and meta-analysis**

**Authors**

Yuki Kotani, Takatoshi Koroki, Yuki Kotani, Takeshi Nomura, Yoshiro Hayashi

**Table of contents**

Search strategy for systematic literature review 3

PRISMA 2020 checklist 5

Table. S2. Risk of bias evaluation 9

Table S3. Peri-intubation hemodynamic management 9

Table S4. Timing of assessing mortality at the longest follow-up 9

Table S5. Sensitivity analyses for secondary outcomes 10

Supplementary Figure S1. Forest plot for peri-intubation cardiovascular instability based on intervention drugs 12

Supplementary Figure S2. Forest plot for peri-intubation cardiovascular instability based on comparator types 12

Supplementary Figure S3. Forest plot for peri-intubation cardiovascular instability based on outcome definition 13

Supplementary Figure S4. Forest plot for peri-intubation cardiovascular instability in low risk-of-bias studies 13

Supplementary Figure S5. Trial sequential analysis for peri-intubation cardiovascular instability 14

Supplementary Figure S6. Forest plot for peri-intubation hypoxemia 14

Supplementary Figure S7. Forest plot for duration of mechanical ventilation 14

Supplementary Figure S8. Forest plot for successful intubation on the first attempt 15

Supplementary Figure S9. Forest plot for intensive care unit length of stay 15

Supplementary Figure S10. Forest plot for mortality at the longest follow-up 15

Supplementary references 16

# Search strategy for systematic literature review

PubMed

"Critical Illness"[Mesh] OR "Intensive Care Units"[Mesh] OR critical care[tiab] OR critically ill[tiab] OR ICU[tiab] OR intensive care[tiab] OR emergency department[tiab] OR emergency setting[tiab] OR rapid sequence[tiab] OR rapid tracheal intubation[tiab]) AND ("Intubation, Intratracheal"[Mesh] OR intubation[tiab] OR "endotracheal intubation"[tiab] OR airway management[tiab] OR intubations[tiab] OR rapid sequence induction[tiab]) AND (randomized controlled trial[pt] OR randomized[tiab] OR randomised[tiab] OR RCT[tiab] OR trial[tiab]) NOT (review[pt] OR editorial[pt] OR letter[pt] OR observational[pt] OR cohort[pt] OR case-control[pt] OR cross-sectional[pt]) NOT ("animals"[MeSH Terms] NOT "humans"[MeSH Terms]) AND (fentanyl[tiab] OR sufentanil[tiab] OR alfentanil[tiab] OR remifentanil[tiab] OR carfentanil[tiab])

Cochrane Library

([mh "Critical Illness"] OR [mh "Intensive Care Units"] OR "critical care":ti,ab,kw OR "critically ill":ti,ab,kw OR ICU:ti,ab,kw OR "intensive care":ti,ab,kw OR "emergency department":ti,ab,kw OR "emergency setting":ti,ab,kw OR "rapid sequence":ti,ab,kw OR "rapid tracheal intubation":ti,ab,kw)

AND

([mh "Intubation, Intratracheal"] OR intubation:ti,ab,kw OR "endotracheal intubation":ti,ab,kw OR "airway management":ti,ab,kw OR intubations:ti,ab,kw OR "rapid sequence induction":ti,ab,kw)

AND

(randomized:ti,ab,kw OR randomised:ti,ab,kw OR RCT:ti,ab,kw OR trial:ti,ab,kw)

Embase

(('critical illness':de OR 'intensive care unit':de OR 'critical care':ti,ab OR 'critically ill':ti,ab OR ICU:ti,ab OR 'intensive care':ti,ab OR 'emergency department':ti,ab OR 'emergency setting':ti,ab OR 'rapid sequence':ti,ab OR 'rapid tracheal intubation':ti,ab)

AND

('endotracheal intubation':de OR intubation:ti,ab OR 'endotracheal intubation':ti,ab OR 'airway management':ti,ab OR intubations:ti,ab OR 'rapid sequence induction':ti,ab)

AND

('randomized controlled trial':it OR randomized:ti,ab OR randomised:ti,ab OR RCT:ti,ab OR trial:ti,ab)

AND

(fentanyl:ti,ab OR sufentanil:ti,ab OR alfentanil:ti,ab OR remifentanil:ti,ab OR carfentanil:ti,ab))

NOT

(review:it OR editorial:it OR letter:it OR (animal:de NOT human:de))

ClinicalTrials.gov

(fentanyl OR sufentanil OR alfentanil OR remifentanil OR carfentanil)

AND

(intubation OR tracheal intubation OR endotracheal intubation OR rapid sequence OR airway management)

AND

(ICU OR critical illness OR critically ill OR intensive care OR emergency OR ED)

WHO ICTRP

(fentanyl OR sufentanil OR alfentanil OR remifentanil OR carfentanil OR opioid)

AND

(intubation OR "tracheal intubation" OR "endotracheal intubation" OR "rapid sequence" OR RSI OR "airway management")

AND

(ICU OR "critical illness" OR "critically ill" OR "intensive care" OR emergency OR "emergency department")

# PRISMA 2020 checklist

| **Section and Topic** | **Item #** | **Checklist item** | **Location where item is reported** |
| --- | --- | --- | --- |
| **TITLE** | | |  |
| Title | 1 | Identify the report as a systematic review. | 1 |
| **ABSTRACT** | | |  |
| Abstract | 2 | See the PRISMA 2020 for Abstracts checklist. | 2, 3 |
| **INTRODUCTION** | | |  |
| Rationale | 3 | Describe the rationale for the review in the context of existing knowledge. | 4,5 |
| Objectives | 4 | Provide an explicit statement of the objective(s) or question(s) the review addresses. | 4,5 |
| **METHODS** | | |  |
| Eligibility criteria | 5 | Specify the inclusion and exclusion criteria for the review and how studies were grouped for the syntheses. | 5,6 |
| Information sources | 6 | Specify all databases, registers, websites, organisations, reference lists and other sources searched or consulted to identify studies. Specify the date when each source was last searched or consulted. | 5 |
| Search strategy | 7 | Present the full search strategies for all databases, registers and websites, including any filters and limits used. | Supplementary materials |
| Selection process | 8 | Specify the methods used to decide whether a study met the inclusion criteria of the review, including how many reviewers screened each record and each report retrieved, whether they worked independently, and if applicable, details of automation tools used in the process. | 6 |
| Data collection process | 9 | Specify the methods used to collect data from reports, including how many reviewers collected data from each report, whether they worked independently, any processes for obtaining or confirming data from study investigators, and if applicable, details of automation tools used in the process. | 7 |
| Data items | 10a | List and define all outcomes for which data were sought. Specify whether all results that were compatible with each outcome domain in each study were sought (e.g. for all measures, time points, analyses), and if not, the methods used to decide which results to collect. | 7,8 |
|  | 10b | List and define all other variables for which data were sought (e.g. participant and intervention characteristics, funding sources). Describe any assumptions made about any missing or unclear information. | 7,8 |
| Study risk of bias assessment | 11 | Specify the methods used to assess risk of bias in the included studies, including details of the tool(s) used, how many reviewers assessed each study and whether they worked independently, and if applicable, details of automation tools used in the process. | 8 |
| Effect measures | 12 | Specify for each outcome the effect measure(s) (e.g. risk ratio, mean difference) used in the synthesis or presentation of results. | 9 |
| Synthesis methods | 13a | Describe the processes used to decide which studies were eligible for each synthesis (e.g. tabulating the study intervention characteristics and comparing against the planned groups for each synthesis (item #5)). | 9 |
|  | 13b | Describe any methods required to prepare the data for presentation or synthesis, such as handling of missing summary statistics, or data conversions. | 9 |
|  | 13c | Describe any methods used to tabulate or visually display results of individual studies and syntheses. | 9 |
|  | 13d | Describe any methods used to synthesize results and provide a rationale for the choice(s). If meta-analysis was performed, describe the model(s), method(s) to identify the presence and extent of statistical heterogeneity, and software package(s) used. | 9 |
|  | 13e | Describe any methods used to explore possible causes of heterogeneity among study results (e.g. subgroup analysis, meta-regression). | 9 |
|  | 13f | Describe any sensitivity analyses conducted to assess robustness of the synthesized results. | 9 |
| Reporting bias assessment | 14 | Describe any methods used to assess risk of bias due to missing results in a synthesis (arising from reporting biases). | 8 |
| Certainty assessment | 15 | Describe any methods used to assess certainty (or confidence) in the body of evidence for an outcome. | 8 |
| **RESULTS** | | |  |
| Study selection | 16a | Describe the results of the search and selection process, from the number of records identified in the search to the number of studies included in the review, ideally using a flow diagram. | 1- |
|  | 16b | Cite studies that might appear to meet the inclusion criteria, but which were excluded, and explain why they were excluded. | Supplementary materials |
| Study characteristics | 17 | Cite each included study and present its characteristics. | 10,11 |
| Risk of bias in studies | 18 | Present assessments of risk of bias for each included study. | 10 |
| Results of individual studies | 19 | For all outcomes, present, for each study: (a) summary statistics for each group (where appropriate) and (b) an effect estimate and its precision (e.g. confidence/credible interval), ideally using structured tables or plots. | 10 |
| Results of syntheses | 20a | For each synthesis, briefly summarise the characteristics and risk of bias among contributing studies. | 10, Supplementary materials |
|  | 20b | Present results of all statistical syntheses conducted. If meta-analysis was done, present for each the summary estimate and its precision (e.g. confidence/credible interval) and measures of statistical heterogeneity. If comparing groups, describe the direction of the effect. | 11, 12, Table 3 |
|  | 20c | Present results of all investigations of possible causes of heterogeneity among study results. | 11, 12, Table 2, Supplementary materials |
|  | 20d | Present results of all sensitivity analyses conducted to assess the robustness of the synthesized results. | 11, 12, Table 2, Supplementary materials |
| Reporting biases | 21 | Present assessments of risk of bias due to missing results (arising from reporting biases) for each synthesis assessed. | Not applicable |
| Certainty of evidence | 22 | Present assessments of certainty (or confidence) in the body of evidence for each outcome assessed. | 11, 12 Table 3 |
| **DISCUSSION** | | |  |
| Discussion | 23a | Provide a general interpretation of the results in the context of other evidence. | 14,15 |
|  | 23b | Discuss any limitations of the evidence included in the review. | 17,18 |
|  | 23c | Discuss any limitations of the review processes used. | 17,18 |
|  | 23d | Discuss implications of the results for practice, policy, and future research. | 15,16,17 |
| **OTHER INFORMATION** | | |  |
| Registration and protocol | 24a | Provide registration information for the review, including register name and registration number, or state that the review was not registered. | 5 |
|  | 24b | Indicate where the review protocol can be accessed, or state that a protocol was not prepared. | 5 |
|  | 24c | Describe and explain any amendments to information provided at registration or in the protocol. | Not applicable |
| Support | 25 | Describe sources of financial or non-financial support for the review, and the role of the funders or sponsors in the review. | 1 |
| Competing interests | 26 | Declare any competing interests of review authors. | 1 |
| Availability of data, code and other materials | 27 | Report which of the following are publicly available and where they can be found: template data collection forms; data extracted from included studies; data used for all analyses; analytic code; any other materials used in the review. | 19 |

**Table S1. Major exclusions and reasons for exclusion**

| Study | Reason for exclusion |
| --- | --- |
| Chung 1992 [1] | Wrong population |
| Khanykin 2013 [2] | Wrong comparator |
| Manickam 2015 [3] | Congress abstract without full-text publication |
| Chaumeron 2020 [4] | Wrong population |
| Bahreini 2021 [5] | Wrong population |
| Paknezhad 2022 [6] | Wrong comparator |
| Zhang 2024 [7] | Wrong comparator |
| Mostafa 2024 [8] | Wrong intervention |
| Chen 2024 [9] | Wrong population |
| NCT05464979 | Trial registration of an included study |
| NCT01823328 | Wrong intervention |
| NCT05014711 | Wrong population |
| NCT02709473 | Wrong population |
| NCT02125201 | Wrong population |
| NCT02029898 | Wrong population |
| ACTRN12621001764820 | Wrong comparator |
| CTRI/2021/08/035397 | Wrong comparator |
| ACTRN12616001570471 | Trial registration of an included study |

# Table. S2. Risk of bias evaluation


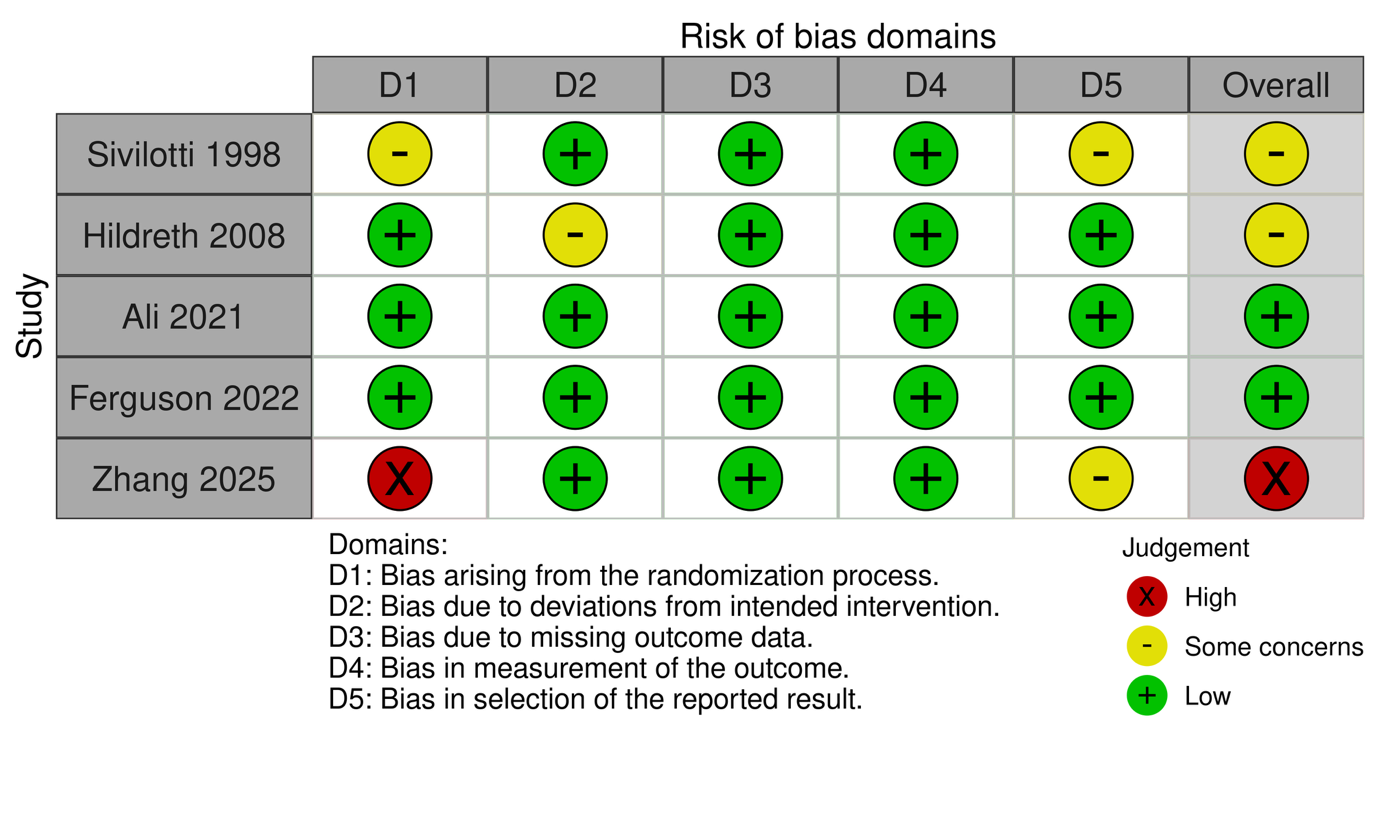


# Table S3. Peri-intubation hemodynamic management

| **Study** | **Peri-intubation hemodynamic management** |
| --- | --- |
| Sivilotti  1998^a^ | Not specified |
| Hildreth  2009 | Not specified |
| Ali  2021 | Norepinephrine was administered to keep mean arterial pressure of 65 mmHg. |
| Ferguson  2022 | Fluid resuscitation and inotropic medications were used when necessary. |
| Zhang  2025 | Norepinephrine was administered to manage hypotension. |

# Table S4. Timing of assessing mortality at the longest follow-up

| **Study** | **Timing** |
| --- | --- |
| Hildreth 2008 | Not specified |
| Ferguson 2022 | 30 days |
| Zhang 2025 | 28 days |

# Table S5. Sensitivity analyses for secondary outcomes

| Subgroup | No. of studies | Fentanyl | Control | Risk ratio or mean difference  (95% CI) | P value | I^2^ | P for interaction |
| --- | --- | --- | --- | --- | --- | --- | --- |
| *Peri-intubation hypoxemia* |  |  |  |  |  |  |  |
| Overall | 2 | 32/171 (19%) | 36/206 (17%) | 0.93 (0.31 to 2.83) | 0.90 | 75% |  |
| Type of comparator |  |  |  |  |  |  | 0.046 |
| Placebo | 1 | 28/143 (20%) | 19/148 (13%) | 1.53 (0.89 to 2.61) | 0.12 | NA |  |
| Non-placebo drug | 1 | 21/88 (24%) | 25/108 (23%) | 0.79 (0.17 to 3.65) | 0.77 | NA |  |
| Low risk-of-bias studies | 1 | 28/143 (20%) | 19/148 (13%) | 1.53 (0.89 to 2.61) | 0.12 | NA |  |
| *Successful intubation on the first attempt* |  |  |  |  |  |  |  |
| Overall | 1 | 132/140 (94%) | 136/148 (92%) | 1.03 (0.96 to 1.09) | 0.42 | NA |  |
| Low risk-of-bias studies | 1 | 132/140 (94%) | 136/148 (92%) | 1.03 (0.96 to 1.09) | 0.42 | NA |  |
| *Duration of mechanical ventilation* |  |  |  |  |  |  |  |
| Overall | 3 |  |  | 0.19 (-4.29 to 4.66) | 0.93 | 89% |  |
| Type of intervention |  |  |  |  |  |  | 0.005 |
| Fentanyl | 2 |  |  | -2.29 (-6.67 to 2.09) | 0.31 | 88% |  |
| Any analogue | 1 |  |  | 6.70 (2.23 to 11.13) | 0.003 | NA |  |
| Type of comparator |  |  |  |  |  |  | 0.04 |
| Placebo | 2 |  |  | 2.84 (-3.98 to 9.66) | 0.41 | 89% |  |
| Non-placebo drug | 1 |  |  | -4.80 (-7.84 to -1.76) | 0.002 | NA |  |
| Low risk-of-bias studies | 1 |  |  | -0.30 (-0.73 to 0.13) | 0.17 | NA |  |
| *ICU length of stay* |  |  |  |  |  |  |  |
| Overall | 2 |  |  | 0.93 (-11.12 to 12.98) | 0.88 | 94% |  |
| Type of intervention |  |  |  |  |  |  | < 0.001 |
| Fentanyl | 1 |  |  | -5.10 (-8.69 to -1.51) | 0.005 | NA |  |
| Any analogue | 1 |  |  | 7.20 (2.25 to 12.15) | 0.004 | NA |  |
| Type of comparator |  |  |  |  |  |  | < 0.001 |
| Placebo | 1 |  |  | 7.20 (2.25 to 12.15) | 0.004 | NA |  |
| Non-placebo drug | 1 |  |  | -5.10 (-8.69 to -1.51) | 0.005 | NA |  |
| *Mortality at the longest follow-up* |  |  |  |  |  |  |  |
| Overall | 3 | 38/204 (19%) | 36/206 (17%) | 0.93 (0.31 to 2.83) | 0.90 | 75% |  |
| Type of intervention |  |  |  |  |  |  | 0.45 |
| Fentanyl | 2 | 27/161 (17%) | 37/160 (23%) | 0.62 (0.18 to 2,11) | 0.44 | 26% |  |
| Any analogue | 1 | 11/43 (26%) | 46/198 (23%) | 1.08 (0.50 to 2.32) | 0.20 | NA |  |
| Type of comparator |  |  |  |  |  |  | 0.23 |
| Placebo | 2 | 38/186 (20%) | 44/186 (24%) | 0.86 (0.59 to 1.27) | 0.45 | 0% |  |
| Non-placebo drug | 1 | 0/18 (0%) | 2/12 (17%) | 0.14 (0.01 to 2.62) | 0.19 | NA |  |
| Low risk-of-bias studies | 1 | 27/143 (19%) | 35/148 (24%) | 0.80 (0.51 to 1.25) | 0.32 | NA |  |

NA, not applicable; ICU, intensive care unit

# Supplementary Figure S1. Forest plot for peri-intubation cardiovascular instability based on intervention drugs

# Supplementary Figure S2. Forest plot for peri-intubation cardiovascular instability based on comparator types

# Supplementary Figure S3. Forest plot for peri-intubation cardiovascular instability based on outcome definition

# Supplementary Figure S4. Forest plot for peri-intubation cardiovascular instability in low risk-of-bias studies

# Supplementary Figure S5. Trial sequential analysis for peri-intubation cardiovascular instability


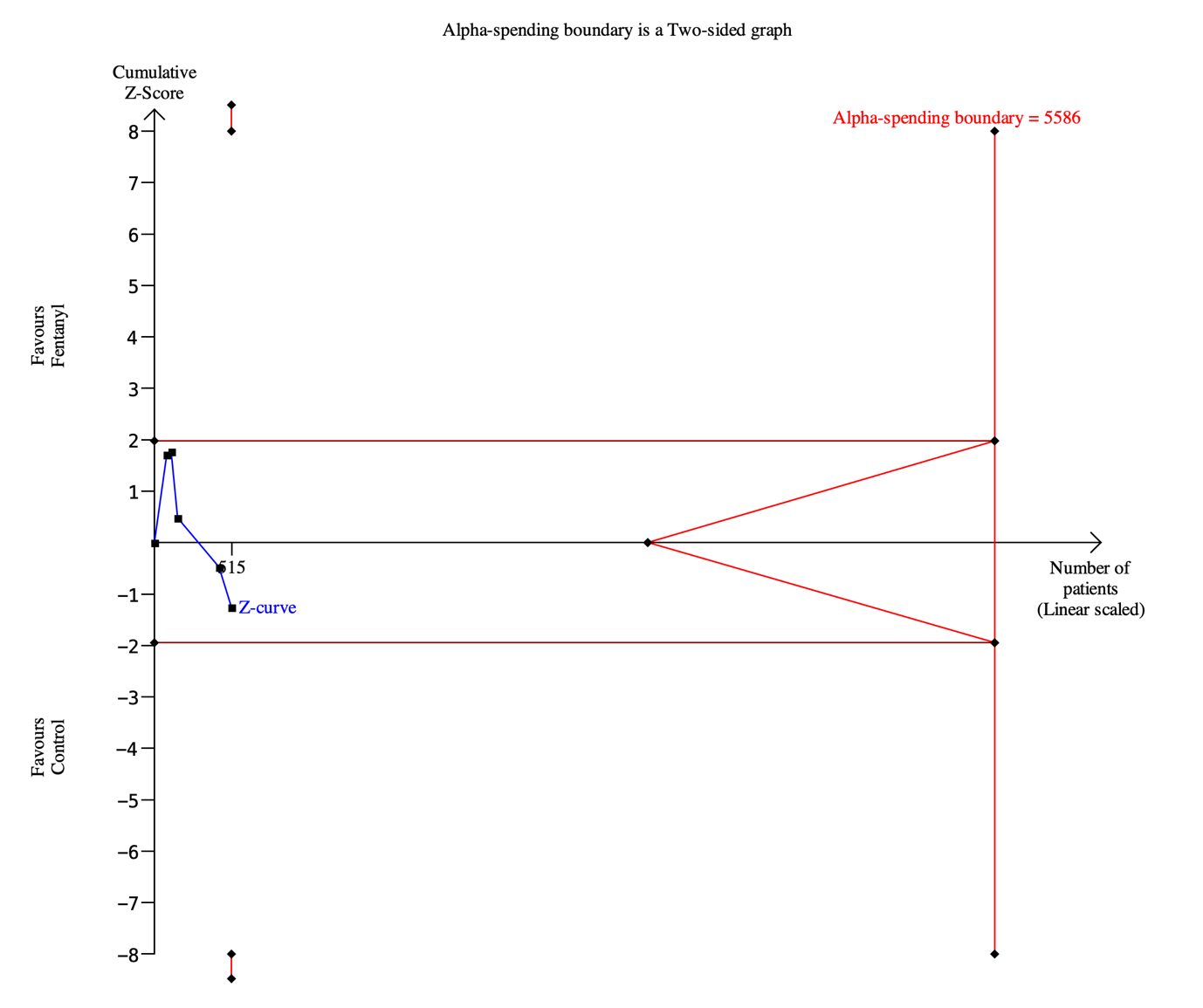


# Supplementary Figure S6. Forest plot for peri-intubation hypoxemia

# Supplementary Figure S7. Forest plot for duration of mechanical ventilation

# Supplementary Figure S8. Forest plot for successful intubation on the first attempt

# Supplementary Figure S9. Forest plot for intensive care unit length of stay

# Supplementary Figure S10. Forest plot for mortality at the longest follow-up

# Supplementary references

1. Chung KS, Sinatra RS, Halevy JD, Paige D, Silverman DG. A comparison of fentanyl, esmolol, and their combination for blunting the haemodynamic responses during rapid-sequence induction. Can J Anaesth. 1992;39:774–9.

2. Khanykin B, Siddiqi R, Jensen PF, Bigler DR, Atroshchenko GV. Comparison of remifentanil and low-dose fentanyl for fast-track cardiac anesthesia: a prospective randomized study. Heart Surg Forum. 2013;16:E324-8.

3. Manickam VS, Mohanasundaram P. Ketofol versus fentofol as induction agents for endotracheal intubation in emergency room. Int J Emerg Med. 2015;8:1–1.

4. Chaumeron A, Castanie J, Fortier LP, Basset P, Bastide S, Alonso S, et al. Efficacy and safety of remifentanil in a rapid sequence induction in elderly patients: A three-arm parallel, double blind, randomised controlled trial. Anaesth Crit Care Pain Med. 2020;39:215–20.

5. Bahreini M, Talebi Garekani M, Sotoodehnia M, Rasooli F. Comparison of the efficacy of ketamine- propofol versus sodium thiopental-fentanyl in sedation: a randomised clinical trial. Emerg Med J. 2021;38:211–6.

6. Paknezhad S, Serati E, Mehdizadeh Esfanjani R, Soleimanpour M, Soleimanpour H. Effects of fentanyl versus remifentanil pretreatment on the QTc interval in patients undergoing rapid sequence intubation: A randomized clinical trial. Anesth Pain Med. 2022;12:e131184.

7. Zhang Y, Miller M, Buttfield A, Burns B, Lawrie K, Gaston J, et al. Alfentanil versus fentanyl for emergency department rapid sequence induction with ketamine: A-FAKT, a pilot randomized trial. Am J Emerg Med. 2024;84:25–32.

8. Mostafa M, Hasanin A, Reda B, Elsayad M, Zayed M, Abdelfatah ME. Comparing the hemodynamic effects of ketamine versus fentanyl bolus in patients with septic shock: a randomized controlled trial. J Anesth. 2024;38:756–64.

9. Chen X, Han M, Shu A, Zhou M, Wang K, Cheng C. Effects of different doses of alfentanil on cardiovascular response to rapid sequence intubation in elderly patients: a parallel-controlled randomized trial. BMC Anesthesiol. 2024;24:290.
